# Incidence of Recurrent Venous Thromboembolism in a Population-Based Cohort

**DOI:** 10.1177/10760296241293337

**Published:** 2024-10-24

**Authors:** Tomas Ruthström, Lovisa Hägg, Lars Johansson, Marcus M Lind, Magdalena Johansson

**Affiliations:** 1 Department of Public Health and Clinical Medicine, Skellefteå Research Unit, 8075 Umeå University, Skellefteå, Sweden

**Keywords:** venous thromboembolism, pulmonary embolism, deep vein thrombosis, recurrence, cancer, mortality, incidence, cohort study, risk factor

## Abstract

The incidence of recurrent venous thromboembolism (VTE) changes over time from the first VTE event and depends on the presence of risk factors. In this study, we aimed to determine the yearly incidence of VTE recurrence during five years of follow-up after a first-ever VTE event. For this cohort study, we identified persons who experienced a validated first-ever VTE between 2006–2014 in northern Sweden. These patients’ medical records were reviewed to identify recurrent VTE events during five years of follow-up. The yearly incidence rates (IRs) of recurrent VTE per 100 person-years were calculated and stratified into three groups defined by characteristics at the first-ever VTE event: no risk factors, cancer, or other risk factors. A total of 1413 persons experienced a first-ever VTE during the study period, of whom 213 experienced a recurrent VTE. Among persons without risk factors, the IR was 4.2 during the first year of follow-up, and 4.1 during the fifth year. Among persons with cancer, the IR was 9.5 during the first year, and 5.4 during the fifth year. Among persons with other risk factors, the corresponding IRs were 6.1 and 2.3. In conclusion, after a first-ever VTE event, persons with cancer had the highest recurrence rate during the first years of follow-up. Among persons with cancer who were alive after five years, the incidence of recurrent VTE during the fifth year was similar to that in participants without risk factors.

## Introduction

Venous thromboembolism (VTE) carries a substantial risk of recurrence, and persons who experience a recurrent VTE event have increased mortality, compared to persons who experience a single VTE without recurrence.^1^ Moreover, recurrent VTEs are costly for the healthcare system.^2^ First-event VTE and recurrent VTE events tend to be of the same type: pulmonary embolism or lower extremity deep vein thrombosis (DVT).^3^ It is often challenging to diagnose a recurrent VTE event–for example, because post-thrombotic changes can be difficult to distinguish from new thrombi.^4,5^ Among persons examined with compression ultrasonography for suspected ipsilateral recurrent DVT, one-third have indeterminate results.^6^

Previous studies report that 6%-8% of persons with VTE experience a recurrence during the year following the first event,^7–10^ with men exhibiting a higher risk of VTE recurrence.^11^ The recurrence rate also changes over time and varies depending on the presence or absence of risk factors at the first VTE event.^12^ Cancer is a major risk factor for VTE.^13^

It has been reported that diagnostic codes from administrative registries can result in overestimation of the number of VTE events.^14^ The positive predictive value of a VTE diagnostic code is numerically lower for recurrent events compared to first events.^15^ Therefore, validation of VTE events is particularly important in studies of VTE recurrences. This issue can have a greater impact on recent studies, since an increasing proportion of persons with VTE are treated in an outpatient setting, in which the diagnostic codes for VTE are less reliable.^16^

To prevent VTE recurrences, it is important to increase our knowledge about which patients have a high risk of recurrent VTE events. Several models have been developed to predict the risk of recurrent VTE.^17–19^ However, they are currently not recommended for use in clinical practice due to their methodological limitations and insufficient predictive accuracy.^20^

In our present population-based cohort study, we aimed to determine the yearly incidence rates of VTE recurrence and all-cause mortality during five years of follow-up after a first-ever VTE event. Our study cohort included only validated cases of VTE. We aimed to determine the incidence of VTE recurrence at different time-points after a first-ever VTE event in three groups of patients: persons without risk factors for VTE, persons with cancer, and persons with other risk factors for VTE.

## Methods

### Study Population

The present study was based on the Venous thromboEmbolism In Northern Sweden (VEINS) study population, which has been previously described.^21^ Briefly, the VEINS population includes participants in the Västerbotten Intervention Programme (VIP)–a health examination and health counselling program that invites all residents of Västerbotten County, the vast majority of which are of Western European ancestry, to health examinations at ages 40, 50, and 60 years.^22^ The program started in 1985, and initially also invited participants aged 30 years. All individuals who had participated in the VIP were followed, and first-ever VTE events were identified.

For the current study, we identified first-ever VTE events experienced by the study population between 2006–2014, by searching the National Patient Registry for relevant ICD-10 diagnostic codes from all inpatient, outpatient, emergency department, and primary healthcare visits in Västerbotten County. Events were manually validated by reviewing medical records and radiology reports.^21,23^ Events were classified into three groups according to location (hierarchically, in cases with multiple simultaneous thrombosis locations), as follows: pulmonary embolism, lower extremity DVT, and other VTE location (upper extremity DVT, abdominal venous thrombosis, or central nervous system venous thrombosis). Persons with a verified DVT and documented symptoms of pulmonary embolism were classified into the pulmonary embolism group.

We excluded participants registered with at least one ICD diagnostic code for VTE prior to the VIP health examination. The study population of the present investigation included participants with a validated first-ever VTE event between January 1, 2006 and September 5, 2014 (*n *= 1413).

### Risk Factors at the First-Ever VTE Event

Data about risk factors for their first-ever VTE event was collected by review of medical records. Information was registered regarding hospitalization, surgery, immobilization for over 48 h, cast therapy, pregnancy, postpartum status, travel for over eight hours, and trauma, all within 60 days prior to the VTE event.

Data regarding all types of cancer, except non-melanoma skin cancer, diagnosed within five years before and 90 days after the VTE event was retrieved from the Swedish Cancer Registry.

Events were hierarchically classified into three groups based on the presence or absence of risk factors at the first-ever VTE event: cancer, other risk factor(s), and no risk factor.

### Follow-up Period

For analyses of VTE recurrence, participants were followed until VTE recurrence. Participants without a VTE recurrence were censored after five years of follow-up. Participants without VTE recurrence who migrated before five years of follow-up had passed were followed until the date of the last entry in the medical records before migration. Participants without VTE who died before five years of follow-up had passed were followed to the date of death. For analyses of mortality, the end of follow-up was the earliest of the following: death, the date of the last entry in the medical record (for persons lost to follow-up due to migration), or five years after the first-ever VTE event.

### Validation and Classification of Recurrent VTE Events

To identify recurrent VTE events, medical records and radiology reports were manually reviewed for five years after the first-ever VTE event. Recurrent events were classified according to the same criteria used for first-ever events, with the additional criterion that a new thrombosis had to be identified radiologically or at autopsy. A VTE recurrence was defined as a thrombosis at a previously uninvolved location, a thrombosis at a previously involved location with documented resolution prior to recurrence, a thrombosis surrounded by contrast material at a previously involved location, or a thrombosis deemed new by the examining radiologist. An event occurring within 29 days of the first-ever event was regarded as an extension of the first-ever event, rather than as a new event.

Recurrent VTE events were further classified into three groups based on location: pulmonary embolism, lower extremity DVT, and other VTE location. Persons who had a verified lower extremity DVT and who were additionally diagnosed within 7 days with a verified pulmonary embolism, or had documented symptoms consistent with pulmonary embolism without radiological examination, were classified as having pulmonary embolism.

Medical records were reviewed to register data regarding the duration of anticoagulant treatment after the first-ever VTE event, and treatment at the recurrent VTE event. The duration of treatment was defined as the time from the first-ever event until the earliest of the following: death, VTE recurrence, cessation of anticoagulant treatment, or five years after the first-ever VTE event.

### Statistical Methods

For descriptive statistics, numbers, proportions, means, and standard deviations were calculated. The chi-squared test and the analysis of variance were used to assess differences between groups. Incidence rates (IRs) for recurrent VTE and death were calculated and expressed per 100 person-years of follow-up, with 95% confidence intervals (CIs). CIs were calculated using the quadratic approximation to the Poisson log likelihood for the log-rate parameter. A Kaplan-Meier curve was drawn to show the Kaplan-Meier failure function.

Univariable and multivariable Cox proportional hazards regression models were used to calculate the hazard ratios (HRs) and 95% CIs for associations between characteristics at the first-ever VTE event and risk of recurrent VTE. The adjustment variables to be included in the multivariable model were prespecified (age, sex, index VTE location, the presence of cancer or other VTE risk factor at the index event and duration of anticoagulation treatment). A total of 16 persons (1.1%) had missing data about treatment duration and were excluded from analyses including this variable. A cumulative hazard function was generated from the multivariable Cox proportional hazards model. In a sensitivity analysis where death was entered as a competing risks event, competing risks-regression based on Fine and Gray's proportional subhazards model was used to explore the association between characteristics at the first-ever VTE event and risk of recurrent VTE. Statistical analyses were performed using SPSS (versions 18 and 25, Armonk, NY, IBM Corp.) and STATA version 14 (Stata Corporation, College Station, TX, USA).

### Ethics

The study was approved by the Regional Ethics Review Board, Umeå, Sweden, approval number 06-162 M §157/06. At the VIP health examination, all participants provided informed consent to participate in research. All participants in the study received a letter providing information about the study, where to obtain further information and were given the option to decline participation.

## Results

The study cohort comprised 1413 persons who experienced a first-ever VTE event between 2006–2014. Table 1 presents the participants’ baseline characteristics by risk factor status at the index event. The mean age at the first-ever VTE event was 66 years, and 53.2% were males. Among the first-ever VTE events, 52.2% were pulmonary embolisms, and 39.0% were lower extremity DVTs. In 509 participants (36.0%), cancer was diagnosed within five years prior to or 90 days after the first-ever VTE event; 403 persons (28.5%) had other risk factors at their first-ever VTE event; and 501 persons (35.5%) had no risk factor at their first-ever VTE event. Other than cancer, hospitalization was the most common risk factor for VTE.

**Table 1.** Characteristics of Cohort by Risk Factor at index (n = 1413).

	No risk factor (n = 501)	Malignancy^a^ (n = 509)	Other risk factor^b^ (n = 403)	*p*-value
Male sex	304 (60.7)	251 (49.3)	197 (48.9)	<0.001
Age at index event (years)	65.4 ± 10.3	66.7 ± 8.7	65.0 ± 10.8	0.152
Treatment duration (months)	15.4 ± 18.6	8.6 ± 12.5	12.1 ± 16.8	<0.001
Time without treatment (months)	33.1 ± 23.3	12.2 ± 20.3	31.6 ± 24.9	<0.001
Data on treatment duration missing	5 (1.0)	6 (1.2)	5 (1.2)	0.133
Index VTE location				<0.001
PE	231 (46.1)	287 (56.4)	219 (54.3)	
DVT	239 (47.7)	150 (29.5)	162 (40.2)	
Other^c^	31 (6.2)	72 (14.1)	22 (5.5)	
Risk factor at index				
None	501 (100.0)	N/A	N/A	-
Malignancy	N/A	509 (100.0)	N/A	-
Other risk factor	N/A	322 (63.3)	403 (100.0)	-
Hospitalization	N/A	301 (59.1)	304 (75.4)	-
Surgery	N/A	131 (25.7)	174 (43.2)	-
Immobilization	N/A	48 (9.4)	126 (31.3)	-
Cast therapy	N/A	5 (1.0)	44 (10.9)	-
Pregnancy	N/A	0 (0.0)	1 (0.2)	-
Postpartum	N/A	0 (0.0)	2 (0.5)	-
Travel by flight	N/A	2 (0.4)	29 (7.2)	-
Trauma	N/A	9 (1.8)	88 (21.8)	-

Continuous variables are presented as mean ± standard deviation, categorical variables as n (%). Percentages are calculated by risk factor group, ie vertically.

Abbreviations: VTE, venous thromboembolism; PE, pulmonary embolism; DVT, lower extremity deep vein thrombosis.

a Any malignancy except non-melanoma skin cancer, diagnosed within 5 years prior to or 90 days after VTE event.

b Hospitalization, surgery, immobilization for more than 48 h, cast therapy, pregnancy, postpartum, travel by flight for more than 8 h, or trauma requiring medical attention. All risk factors within 60 days prior to VTE event.

c Veins of abdomen, deep veins of upper extremity, or veins of central nervous system.

### VTE Recurrence

The total follow-up time when analyzing VTE recurrence was 4369.2 person-years. At the end of five years of follow-up, 213 participants (15.1%) had experienced a recurrent VTE. Male sex was more common among participants with recurrent VTE who had no risk factor for VTE at their index event (67.1% men) compared to participants with malignancy at their first event (57.9% men) and participants with other risk factors for VTE at their index event (40.8% men), *p *= 0.01 for differences between groups. Among participants with malignancy at the index event, 45.3% were taking anticoagulants when experiencing a recurrent event, compared to 11.5% of participants without an identified risk factor for VTE at the index event and 17.5% of participants with other risk factors for VTE at the index event. The mean time from index event to VTE recurrence was 2.4 years (SD 1.4) for participants without risk factor for VTE at the index event, 1.6 years (SD 1.3) for participants with malignancy at the index event and 1.9 years (SD 1.5) for participants with another risk factor for VTE at the index event.

The overall IR of recurrent VTE was 4.9 (95% CI 4.3-5.6) per 100 person-years. Among the three participant groups, the IR of recurrent VTE was 8.6 (95% CI 6.9-10.7) per 100 person-years for individuals with cancer at the first-ever event, 3.3 (95% CI 2.5-4.4) for individuals with other risk factors at the first-ever event, and 4.4 (95% CI 3.5-5.4) for individuals with no risk factor at the first-ever event.

**Figure 1.**
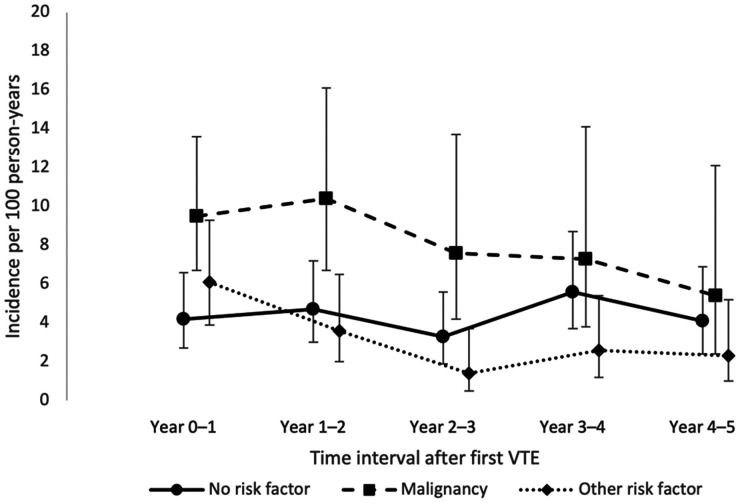
Incidence rates of recurrent venous thromboembolism (VTE) in different time intervals after first VTE, by risk factor at index.

**Figure 2.**
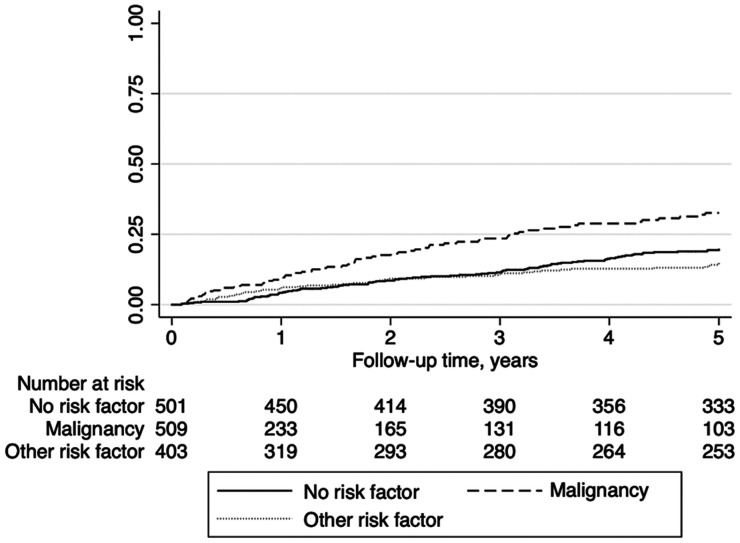
Kaplan-Meier failure function for venous thromboembolism (VTE) recurrence stratified by risk factor status at the index VTE event.

The IRs of recurrent VTE for each of the five years of follow-up are shown in Table 2 and Figure 1. Among persons without risk factors, the IR was 4.2 (95% CI 2.7-6.6) per 100 person-years during the first year of follow-up, and 4.1 (95% CI 2.4-6.9) during the fifth year. Among persons with cancer, the IR was 9.5 (95% CI 6.7-13.6) during the first year, and 5.4 (95% CI 2.4-12.1) during the fifth year. Among persons with other risk factors, the corresponding IRs were 6.1 (95% CI 3.9-9.3) and 2.3 (95% CI 1.0-5.2). A graph showing the Kaplan-Meier failure function for VTE recurrence stratified by risk factor status at index is shown in Figure 2.

**Table 2.** Number of Occurrences, Number at Risk, and Incidence Rates of Recurrent VTE in Different Time Intervals After First VTE, by Risk Factor at index.

Group	Key	Year 0-1	Year 1-2	Year 2-3	Year 3-4	Year 4-5
No risk factor (n = 501)	*n*/at risk IR (95% CI)	20/501 4.2 (2.7-6.6)	20/450 4.7 (3.0-7.2)	13/414 3.3 (1.9-5.6)	21/390 5.6 (3.7-8.7)	14/356 4.1 (2.4-6.9)
Malignancy^a^ (n = 509)	*n*/at risk IR (95% CI)	30/509 9.5 (6.7-13.6)	20/233 10.4 (6.7-16.1)	11/165 7.6 (4.2-13.7)	9/131 7.3 (3.8-14.1)	6/116 5.4 (2.4-12.1)
Other risk factor^b^ (n = 403)	*n*/at risk IR (95% CI)	21/403 6.1 (3.9-9.3)	11/319 3.6 (2.0-6.5)	4/293 1.4 (0.5-3.7)	7/280 2.6 (1.2-5.4)	6/264 2.3 (1.0-5.2)

Abbreviations: VTE, venous thromboembolism; IR, incidence rate (per 100 person-years); CI, confidence interval.

a Any malignancy except non-melanoma skin cancer, diagnosed within 5 years prior to or 90 days after VTE event.

b Hospitalization, surgery, immobilization for more than 48 h, cast therapy, pregnancy, postpartum, travel by flight for more than 8 h, or trauma requiring medical attention. All risk factors within 60 days prior to VTE event.

Table 3 shows the associations between characteristics at the first-ever VTE event and risk of VTE recurrence. Cancer at the first-ever VTE event was associated with a higher risk of VTE recurrence (HR 1.81, 95% CI 1.31-2.50) compared to no risk factor at the first VTE event. Age, sex, and location of the first-ever VTE event were not associated with risk of recurrence. A longer treatment duration was associated with a lower recurrence risk. A cumulative hazard function derived from the multivariable Cox proportional hazards model is presented in Supplemental Material Figure 1. When the analysis was stratified for no risk factors, cancer, and other risk factors at the first-ever VTE event, neither age, sex, nor VTE location were significantly associated with risk of VTE recurrence in any of the strata in a fully adjusted model. We conclude that the main determinant of risk of recurrent VTE in the present study is the presence of cancer at the index VTE event.

**Table 3.** Predictors of Venous Thromboembolism Recurrence.

	Person-years	Recurrent VTE	Incidence rate^d^	Univariable model		Multivariable model	
				Hazard Ratio (95% CI)	P-value	Hazard Ratio (95% CI)	P-value
Male sex	2437.8	123	5.0 (4.2-6.0)	1.08 (0.83-1.42)	0.57	1.18 (0.89-1.55)	0.25
Age at index event (years)	4369.2	213	4.9 (4.3-5.6)	1.01 (0.995-1.02)	0.21	1.01 (0.998-1.03)	0.08
Index VTE location							
DVT	1879.6	100	5.3 (4.4-6.5)	1 (ref.)		1 (ref.)	
PE	2192.3	92	4.2 (3.4-5.1)	0.78 (0.59-1.04)	0.09	0.97 (0.72-1.31)	0.85
Other^a^	297.2	21	7.1 (4.6-10.8)	1.30 (0.81-2.09)	0.27	1.11 (0.68-1.80)	0.68
Index risk factor							
No risk factor	2015.0	88	4.4 (3.5-5.4)	1 (ref.)		1 (ref.)	
Malignancy^b^	885.4	76	8.6 (6.9-10.7)	1.89 (1.39-2.58)	<0.001	1.81 (1.31-2.50)	<0.001
Other risk factor^c^	1468.8	49	3.3 (2.5-4.4)	0.77 (0.54-1.09)	0.14	0.76 (0.53-1.08)	0.12
Treatment duration (months)	4328.1	208	4.8 (4.2-5.5)	0.96 (0.94-0.97)	<0.001	0.95 (0.94-0.97)	<0.001

Abbreviations: CI, confidence interval; VTE, venous thromboembolism; DVT, lower extremity deep vein thrombosis; PE, pulmonary embolism.

a Veins of abdomen, deep veins of upper extremity, or veins of central nervous system.

b Any malignancy except non-melanoma skin cancer, diagnosed within 5 years prior to or 90 days after VTE event.

c Hospitalization, surgery, immobilization for more than 48 h, cast therapy, pregnancy, postpartum, travel by flight for more than 8 h, or trauma requiring medical attention. All risk factors within 60 days prior to VTE event.

d Incidence rate of recurrent VTE per 100 person-years.

### All-Cause Mortality

Among 1413 individuals with a first-ever VTE event, 600 died within five years, corresponding to an IR of 12.6 (95% CI 11.6-13.6) deaths per 100 person-years. Among the 509 individuals with cancer at the first-ever VTE event, 380 died, corresponding to an IR of 38.4 (95% CI 34.8-42.5) deaths per 100 person-years. Among 403 individuals with other risk factors for VTE at the first-ever VTE event, 119 died, corresponding to an IR of 7.6 (95% CI 6.3-9.0) deaths per 100 person-years. Among 501 individuals with no risk factors for VTE at the first-ever VTE event, 101 died, corresponding to an IR of 4.6 (95% CI 3.8-5.6) deaths per 100 person-years.

**Figure 3.**
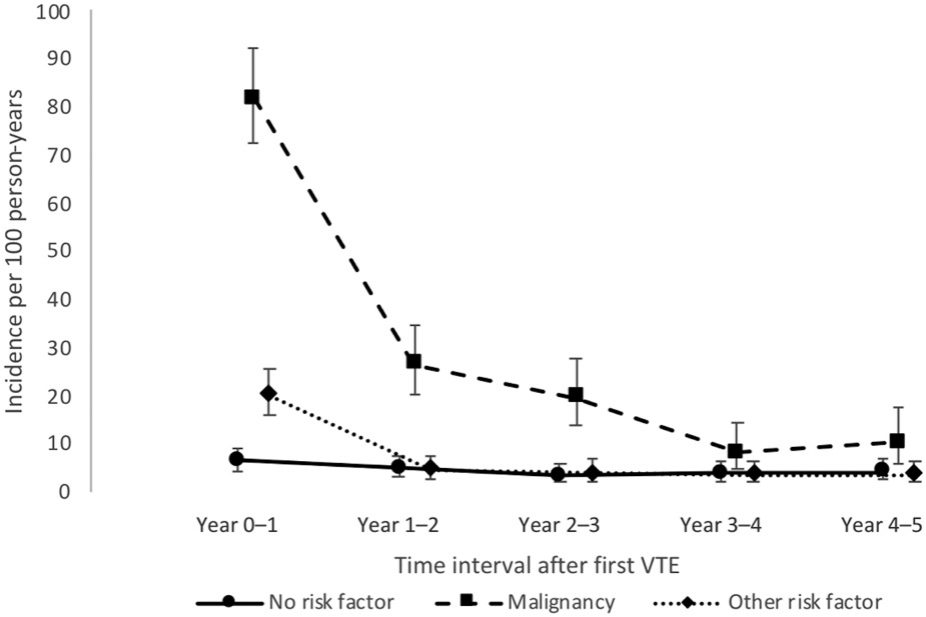
Incidence rates of all-cause mortality in different time intervals after first venous thromboembolism (VTE) event, by risk factor at index.

All-cause mortality rates for each of the five years of follow-up are shown in Table 4 and Figure 3. Mortality rates were highest among persons with cancer, especially during the first year after the first-ever VTE event. Among individuals with cancer or other risk factors at the first-ever VTE event, the mortality rates decreased over time. In contrast, the mortality rates remained constant among individuals with no risk factors at the first-ever VTE event.

**Table 4.** Number of Occurrences, Number at Risk, and Incidence Rates of all-Cause Mortality in Different Time Intervals After First VTE, by Risk Factor at index.

Group	Key	Year 0-1	Year 1-2	Year 2-3	Year 3-4	Year 4-5
No risk factor (n = 501)	*n*/at risk IR (95% CI)	31/501 6.5 (4.5-9.2)	22/469 4.8 (3.2-7.3)	15/447 3.4 (2.1-5.7)	16/431 3.8 (2.3-6.2)	17/415 4.2 (2.6-6.7)
Malignancy^a^ (n = 509)	*n*/at risk IR (95% CI)	265/509 81.6 (72.3-92.0)	56/244 26.5 (20.4-34.5)	33/188 19.6 (13.9-27.6)	12/155 8.1 (4.6-14.2)	14/143 10.3 (6.1-17.4)
Other risk factor^b^ (n = 403)	*n*/at risk IR (95% CI)	71/403 20.1 (15.9-25.4)	15/332 4.6 (2.8-7.7)	12/317 3.9 (2.2-6.8)	11/305 3.7 (2.0-6.6)	10/294 3.5 (1.9-6.4)

Abbreviations: VTE, venous thromboembolism; IR, incidence rate (per 100 person-years); CI, confidence interval.

a Any malignancy except non-melanoma skin cancer, diagnosed within 5 years prior to or 90 days after VTE event.

b Hospitalization, surgery, immobilization for more than 48 h, cast therapy, pregnancy, postpartum, travel by flight for more than 8 h, or trauma requiring medical attention. All risk factors within 60 days prior to VTE event.

### Sensitivity Analyses

In a sensitivity analysis, we defined cancer at the index VTE as cancer diagnosed within two years prior to or 90 days after the VTE event. This analysis yielded results similar to those of the main analysis (data not shown). In a further sensitivity analysis, we performed a competing risks analysis of the association between characteristics at the first-ever VTE event and risk of VTE recurrence where death was considered a competing risks event. The results of this analysis are presented in Supplemental Material Table 1.

## Discussion

Among persons with no risk factors for VTE, the incidence of VTE recurrence was relatively constant over time. In contrast, among persons with cancer or another risk factor for VTE at their first-ever VTE, we observed a decline in the incidence of VTE recurrence over the five years of follow-up. This decline was most pronounced in persons with cancer. Compared to in persons without risk factors for VTE at their first-ever VTE, the risk of VTE recurrence was higher in persons with cancer (HR 1.81, 95% CI 1.31-2.50).

Our finding of a high incidence of VTE recurrence among persons with cancer is in line with the results of previous studies.^8,9,12,24,25^ After the first two years, the risk of VTE recurrence was similar between participants with cancer and participants without risk factors for VTE at their first-ever event. This was also found in a registry-based Danish study.^12^ In our study, it is likely that the participants with the highest risk of VTE recurrence also had more advanced cancers and thus a high early mortality rate. This leads to an issue of competing risks, in that more patients with cancer and a lower recurrence risk remained in the study population, which may explain the decrease of recurrence rates over time among participants with cancer. It has previously been demonstrated that the higher risk of recurrent VTE among persons with cancer is attenuated when accounting for the competing risk of death.^8^ In our study, over half of the study participants with cancer died within the first year after their first-ever VTE event. This is in agreement with the results of two Norwegian studies.^8,26^ It has previously been reported that early after VTE, the high mortality among persons with cancer is driven by the extent of the cancer itself, rather than by the severity of the VTE event.^24^

In our study, 36.0% of persons with a first-ever VTE event had cancer. Previous studies have reported that cancer-related VTE constituted between 20%-30% of VTEs,^8,24,27^ or lower proportions.^7,12,28^ Notably, the method of patient inclusion, for example, including patients at outpatient visits at specialist coagulation clinics, can lead to underrepresentation of severely ill patients (eg persons with metastatic cancer), thereby decreasing the external validity of the findings. Due to our use of the VIP health examinations, our study population included a representative sample of the population of the area.^29^ Therefore, it is likely that our study population comprised a representative proportion of persons with cancer.

Among participants without cancer at the first-ever VTE event, the participants with another risk factors for VTE did not have a significantly lower risk of recurrence during the five years of follow-up, compared to those who had no risk factor for VTE at their first-ever VTE event. This finding is corroborated by some previous studies,^9^ but not all.^12,30^ The differences in study results may be explained by the varying definitions of risk factors for VTE. For example, one study included conditions such as obesity in their definition of risk factors for VTE,^12^ and the definition of active cancer can differ from that used in our present study.^30^ Moreover, in registry-based studies,^12^ data regarding risk factors for VTE are collected using diagnostic codes, such that correct classification of VTE events as provoked or unprovoked depends on the accuracy of the administrative registries. Notably, the positive predictive value of a diagnostic code has been shown to vary widely between different medical conditions.^31^

The International Society on Thrombosis and Haemostasis has divided VTE risk factors into three groups (minor transient [eg estrogen therapy], major transient [eg major surgery] and persistent [eg cancer]).^13^ The VTE recurrence risk after a VTE event provoked by a minor transient risk factors seems to be higher than that after one provoked by a major transient risk factor.^32^ Possibly, differences in the recurrence rate after a provoked VTE event could at least partly be due to between-study differences in the proportion of events provoked by major and minor transient risk factors.

In this study, VTE location was not associated with risk of recurrence. As the distribution of VTE locations differed substantially between the three groups based on presence or absence of risk factors for VTE, we also analyzed the three groups separately and did not find an association between VTE location and recurrence risk in any of the groups with cancer, other risk factors for VTE and no risk factors for VTE. Our finding is in agreement with some previous studies^9,30,33,34^ and in contrast to others where VTE recurrence was more common after an initial DVT^3^ or pulmonary embolism.^35^

We saw no association between age and risk of recurrence in the studied age range, which is in accordance with the results of some previous studies.^36,37^ Other larger studies have found an association between higher age and increased risk of VTE recurrence.^9,38,39^ It is possible that our finding of no association between age and risk of recurrence is due to insufficient power.

In our study, we found no association between sex and risk of VTE recurrence. Previous research has shown either no difference in recurrence risk between the sexes^7,9,40^ or higher recurrence rates in men.^27,41^ A higher recurrence rate in men was also seen in a study where recurrence rates after a first VTE were compared between men and women without reproductive risk factors for VTE.^11^ It is hard to explain the discrepancy of findings between our study and those finding an association between male sex and higher recurrence risk. However, we included both men and women using the same criteria. Also, participation rates in the VIP were similar for men and women. As male sex is included as a predictor for increased risk of VTE recurrence in many prediction models,^17–19^ we find it important to underline that male sex is not associated with increased risk of recurrence in all populations. As our study is of recent date, including persons with first-ever VTE diagnosed between 2006 and 2014, and is performed in Sweden where out-of-pocket expenses for health care are negligible, we think that men and women in our study had approximately equal access to health care and modern diagnostic methods. If women had less access to these factors in older studies, eg due to lower income or gender discrimination, recurrence rates in women may have been underestimated.

The probability of selection bias affecting the results was low in the present study because of the population-based cohort design employed. About 60% of the population in relevant age-groups participated in the VIP, and differences between participants and non-participants were small.^29^ The study was conducted in a recent time-period and is thus unlikely to be subjected to outdated clinical practices regarding the diagnosis and treatment of VTE. Furthermore, as potential VTE events were manually validated, the risk of overestimating the incidence of recurrent VTE was low.

In the present study, all VTE recurrences were included. In contrast, some studies have focused on the recurrence rate after discontinuation of anticoagulant treatment.^42^ As the vast majority of persons with VTE have at least 90 days of anticoagulant treatment,^43,44^ excluding persons with ongoing anticoagulant treatment leads to an underestimation of early recurrences. Furthermore, studies conducted at specialized coagulation clinics can be affected by selection bias as it can be expected that, for instance, VTE patients with metastatic cancer are not included.

The years during which a study was conducted can also influence recurrence rates.^45,46^ For example, changes in use of VTE prophylaxis^47,48^ and a decrease in cancer mortality for most types of cancer^49^ mean that VTE recurrence rates are prone to change over time. We consider our contemporary study to reflect the current incidence of VTE recurrence.

There are some limitations to the present study. Our study population did not include participants under 30 years of age. As a result, it is likely that VTE events related to pregnancy, postpartum and contraceptives containing estrogen are underrepresented in the study population. However, the incidence of VTE before the age of 30 is very low,^23^ making this unlikely to have a large effect on the results of the present study beyond limiting the generalizability to VTE events associated with reproductive factors. Furthermore, data on risk factors, except data on the presence of cancer, were collected from medical records and may have been incomplete in some cases. On the other hand, we consider data on cancer to be accurate due to the high coverage rate and accuracy of the Swedish Cancer Registry (coverage rate estimated at 96% in 1998).^50^

## Conclusions

In conclusion, after a first-ever VTE event, persons with cancer had the highest recurrence rate during the first years of follow-up. Among those with cancer who were alive after five years the incidence of recurrent VTE was similar to that among those with no risk factors for VTE at the first event. Persons with other risk factors for VTE initially had an intermediate incidence of recurrent VTE that thereafter declined to a low level.

## Supplemental Material

sj-docx-1-cat-10.1177_10760296241293337 - Supplemental material for Incidence of Recurrent Venous Thromboembolism in a Population-Based Cohort Supplemental material, sj-docx-1-cat-10.1177_10760296241293337 for Incidence of Recurrent Venous Thromboembolism in a Population-Based Cohort by Tomas Ruthström, Lovisa Hägg, Lars Johansson, Marcus M Lind and Magdalena Johansson in Clinical and Applied Thrombosis/Hemostasis
