# Supplementary material for: Incidence of Recurrent Venous Thromboembolism in a Population-Based Cohort
Source: Clin Appl Thromb Hemost. 2024 Oct 24;30:10760296241293337. doi: 10.1177/10760296241293337 (PMC11523152; doi:10.1177/10760296241293337)
Supplement: sj-docx-1-cat-10.1177_10760296241293337 - Supplemental material for Incidence of Recurrent Venous Thromboembolism in a Population-Based Cohort [file sj-docx-1-cat-10.1177_10760296241293337.docx]

Supplemental Material Figure 1. Cumulative hazard function generated from a multivariable analysis showing the cumulative hazard for recurrent venous thromboembolism by risk factor status at index.


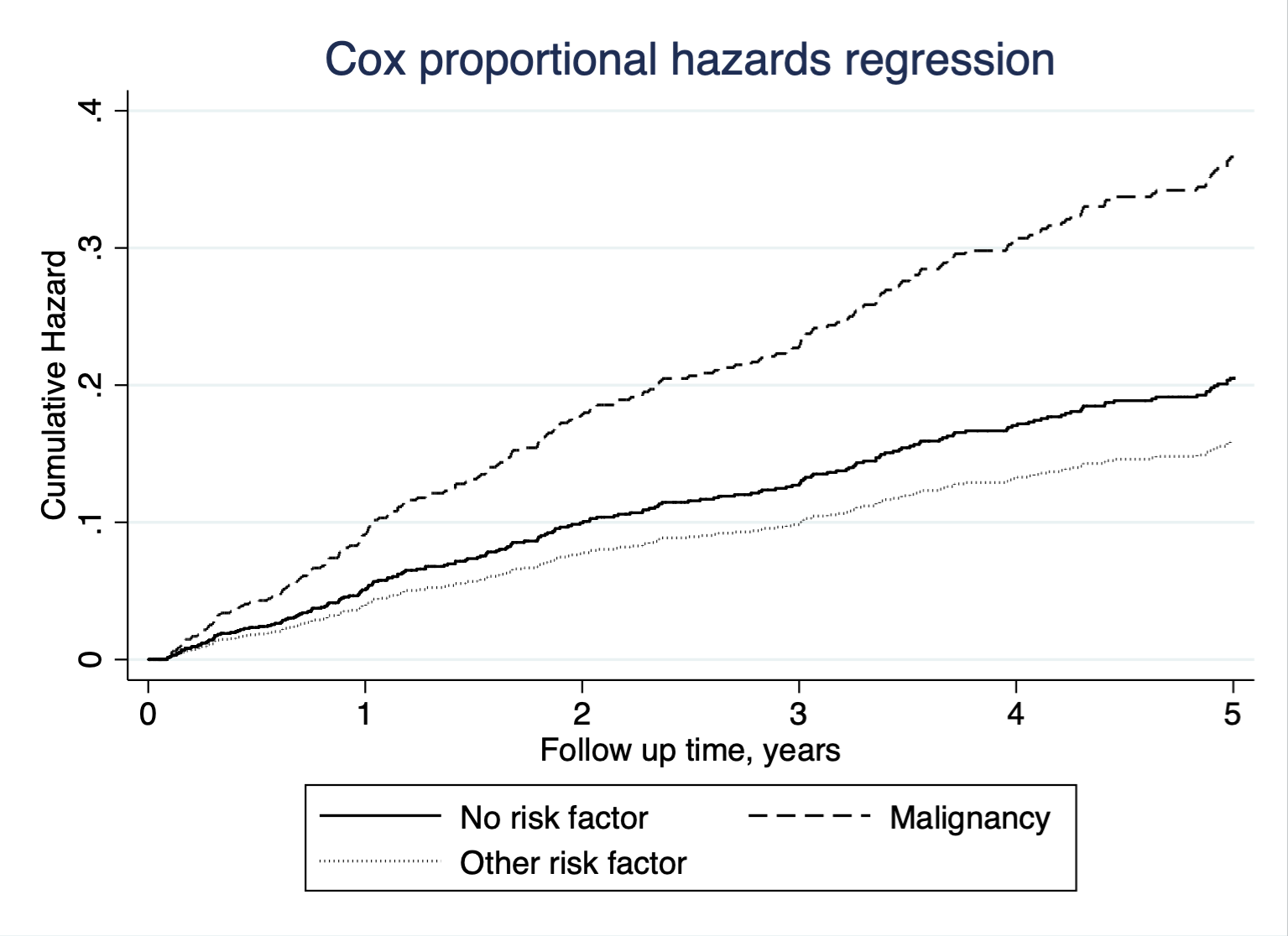


The multivariable analysis of the association between risk factor status at index and risk of recurrent venous thromboembolism was adjusted for sex, age at index event, index VTE location and anticoagulant treatment duration. Malignancy at index was defined as any malignancy except non-melanoma skin cancer, diagnosed within 5 years prior to or 90 days after VTE event. Other risk factor at index was defined as presence of hospitalization, surgery, immobilization for more than 48 hours, cast therapy, pregnancy, postpartum, travel by flight for more than 8 hours, or trauma requiring medical attention within 60 days prior to VTE event.

Supplemental Material Table 1. Predictors of recurrence, multivariable competing risks analysis

| Variable | Subdistribution Hazard Ratio  (95% CI) |
| --- | --- |
| Male sex | 1.23 (0.93–1.63) |
| Age at index event (years) | 1.00 (0.99–1.01) |
| Index VTE location |  |
| - DVT | 1 (ref.) |
| - PE | 0.79 (0.59–1.07) |
| - Otherᵃ | 0.99 (0.61–1.62) |
| Index risk factor |  |
| - No risk factor | 1 (ref.) |
| - Malignancyᵇ | 0.79 (0.57–1.10) |
| - Other risk factorᶜ | 0.70 (0.49–0.99) |
| Anticoagulant treatment duration (months) | 0.98 (0.97–0.99) |

Abbreviations: CI, confidence interval; VTE, venous thromboembolism; DVT, lower extremity deep vein thrombosis; PE, pulmonary embolism

The multivariable analysis was adjusted for sex, age at index event, index VTE location, risk factor status at index and anticoagulant treatment duration.

ᵃ = Veins of abdomen, deep veins of upper extremity, or veins of central nervous system.

ᵇ = Any malignancy except non-melanoma skin cancer, diagnosed within 5 years prior to or 90 days after VTE event.

ᶜ = Hospitalization, surgery, immobilization for more than 48 hours, cast therapy, pregnancy, postpartum, travel by flight for more than 8 hours, or trauma requiring medical attention. All risk factors within 60 days prior to VTE event.
